# Seroprevalence and Risk Factors of the Bluetongue Virus in Cattle in China From 1988 to 2019: A Comprehensive Literature Review and Meta-Analysis

**DOI:** 10.3389/fvets.2020.550381

**Published:** 2021-01-28

**Authors:** Qing-Long Gong, Qi Wang, Xue-Yao Yang, Dong-Li Li, Bo Zhao, Gui-Yang Ge, Ying Zong, Jian-Ming Li, Xue Leng, Kun Shi, Fei Liu, Rui Du

**Affiliations:** ^1^College of Animal Science and Technology, Jilin Agricultural University, Changchun, China; ^2^College of Chinese Medicine Materials, Jilin Agricultural University, Changchun, China

**Keywords:** bluetongue virus, cattle, meta-analysis, China, seroprevalence

## Abstract

**Background:** Bluetongue caused by the bluetongue virus (BTV) is a non-contagious and an insect-borne disease mainly affecting domestic and wild ruminants. Bluetongue in cattle is associated with vesicular lesions, weight loss, low milk production, and low reproductive capacity. It should not be ignored as it is associated with large economic losses to the livestock breeding industry in China. Although many studies have investigated bluetongue virus infection in cattle, no nationwide study on the prevalence of bluetongue virus infection in cattle from China has yet been conducted. This meta-analysis aimed to evaluate the seroprevalence and risk factors for bluetongue in cattle.

**Results:** We collected 50 publications from 1988 to 2019 through PubMed, ScienceDirect, Chinese Web of Knowledge (CNKI), VIP Chinese journal database, and Wanfang database. A total of the pooled bluetongue seroprevalence of 12.2% (5,332/87,472) in cattle was tested. The point estimate of bluetongue collected from 2001 to 2011 was 22.5% (95% CI: 1.2–58.9), which was higher than after 2012 (9.9%, 95% CI: 3.3–19.4). The analysis of the feeding model subgroup revealed that the seroprevalence of bluetongue was significantly higher (*P* < 0.05) among free-range cattle (22.5%; 95% CI: 7.7–42.3) than among cattle from intensive farming systems (1.8%; 95% CI: 0.0–6.7). The seroprevalence of bluetongue in different species showed significant variation (*P* < 0.05), with the highest seroprevalence of 39.8% (95% CI: 18.7–63.0) in buffalo and the lowest seroprevalence of 4.3% (95% CI: 1.2–9.0) in yak. In the zoogeographical division subgroup, the seroprevalence of bluetongue correlated positively within a certain range with the species distribution of *Culicoides*.

**Conclusion:** Our findings suggested that bluetongue was prevalent in cattle in China. In addition, the contact with sheep, other ruminants, or transmission media such as *Culicoides* may increase the seroprevalence of bluetongue disease in cattle. It is necessary to carry out continuous monitoring of the bluetongue seroprevalence. Moreover, comprehensive and improved strategies and measures should be implemented to prevent and control the spread of bluetongue.

## Highlights

- Bluetongue (BT) is highly prevalent and unevenly distributed among Chinese cattle.
- The animal disease prevention plan may effectively reduce this epidemic in cattle.
- An intensive farming model may play an active role in the prevention of BT in cattle.
- This is the first meta-analysis of BT seroprevalence in cattle in China.

## Introduction

Bluetongue caused by the bluetongue virus (BTV) is an arboviral non-contagious disease of domestic and wild ruminants and is designated as a reportable disease by the World Organization for Animal Health (OIE) (1). BTV is a segmented double-stranded (dsRNA) virus belonging to the genus *Orbivirus* of the family Reoviridae and transmitted through biting by hematophagous midges of *Culicoides* (2). BTV is widely prevalent in sheep, goats, cattle, camels, deer, and antelopes. Clinical presentation ranges from asymptomatic to mild fever, salivation, depression, dyspnea, and even abortion and death (2–4), leading to severe economic repercussions for livestock breeding due to direct (miscarriage, mortality, and reduced milk production) or indirect losses (vector control and animal trade restrictions) (5, 6).

Cattle are usually asymptomatic carriers after being infected with BTV, which leads to BTV spreading easily in the herd and being taken lightly. Asymptomatic carrier cattle can be a potential virus reservoir and source of infection on the farm, spreading BTV to the herd mainly through the bites of biological vectors such as *Culicoides* (7), but direct transmission (transplacental or sexual) has also been observed (8), which renders the eradication of BTV difficult.

A total of 27 BTV serotypes have been characterized to date (9–12), as well as other serotypes have been discovered (one in China) (13). The disease outcome varies based on the involved serotype and species (14). In the past decade, bluetongue has spread worldwide. There was a bluetongue pandemic in many Mediterranean and African countries such as Israel, Morocco, Tunisia, and Algeria. Tunisia had an epidemic of bluetongue in 2000 and 2004, and BTV serotype 4 (BTV-4) infection caused bluetongue in Morocco in 2004 (15). Since 1998, Europe has faced sporadic incursions of BTV from other areas. With more frequent invasions of bluetongue, the bluetongue epidemiology has changed dramatically, resulting in cases in many countries where they have never been seen before, such as in Balkan countries, France (Corsica), and Italy (5, 16, 17). BTV-8 was first detected in Europe in 2006; BTV-1 was detected in Spain, Portugal, and southwest France in 2007 (5); and BTV-6 was detected in the Netherlands and Germany (18). Bluetongue caused by BTV-4 infection has occurred successively in Greece, Romania, and Turkey (19).

China is a big cattle-breeding country. According to statistics, 49,292,000 cattle were bred in China in 2014 (20). After the first confirmation of bluetongue disease in Yunnan Province in 1979, BTV-positive livestock were detected in 29 Chinese provinces, including Hubei, Anhui, Sichuan, and Gansu. Of these, 164,576 cattle were investigated from these provinces and the positive rate was 7.4% (21). However, to our knowledge, there is no sufficient systematic analysis about the overall seroprevalence of bluetongue in China. Therefore, this meta-analysis aimed to estimate the seroprevalence of bluetongue in cattle in China. This systematic review and meta-analysis aimed to analyze the pooled seroprevalence of BTV in cattle in China and to assess potential risk factors associated with bluetongue seroprevalence.

## Materials and Methods

### Search Strategy and Selection Criteria

PRISMA was used to report the results in our systematic reviews and meta-analysis (22, 23). We searched the VIP Chinese journal database, Chinese Web of Knowledge (CNKI), Wanfang database, PubMed, and ScienceDirect for papers published in English or Chinese from inception to February 8, 2020. We aimed to screen all English or Chinese published papers on the prevalence of bluetongue in cattle in China. We attempted to contact the authors of the studies that could not be downloaded from the databases for additional information. No attempt was made to identify unpublished reports.

In the PubMed database, the Boolean operator “AND” was used to connect the theme words, and the Boolean operator “OR” was used to connect the free words. We used the theme word “cattle” [Mesh] and the free words “*Bos indicus*,” “zebu,” “zebus,” “*Bos taurus*,” “Cow, Domestic,” “Cows, Domestic,” “Domestic Cow,” “Domestic Cows,” “*Bos grunniens*,” “Yak,” and “Yaks,” which constitute retrieval formula A:

**Table:** 

(“Cattle” [Mesh] OR *Bos indicus* OR Zebu OR Zebus OR *Bos taurus* OR Cow, Domestic OR Cows, Domestic OR Domestic Cow OR Domestic Cows OR *Bos grunniens* OR Yak OR Yaks)
The theme word “bluetongue” [Mesh] and the free words “Blue Tongue” and “Tongue, Blue” constitute retrieval formula B:
(“Bluetongue”[Mesh] OR Blue Tongue OR Tongue, Blue)
The theme word “China” [Mesh] and “People's Republic of China,” “Mainland China,” “Manchuria,” “Sinkiang,” and “Inner Mongolia” constitute retrieval formula C:
(“China” [Mesh] OR People's Republic of China OR Mainland China OR Manchuria OR Sinkiang OR Inner Mongolia)
Finally, formulae A, B, and C were connected with the Boolean operator “AND,” and the final search formula was
(“Bluetongue”[Mesh] OR Blue Tongue) OR Tongue, Blue) AND (“Cattle”[Mesh] OR *Bos indicus* OR Zebu OR Zebus OR Bos taurus OR Cow, Domestic OR Cows, Domestic OR Domestic Cow OR Domestic Cows OR *Bos grunniens* OR Yak OR Yaks) AND (“China”[Mesh] OR People's Republic of China OR Mainland China OR Manchuria OR Sinkiang OR Inner Mongolia)

In the ScienceDirect database, the keywords “bluetongue,” “cattle,” “epidemiology,” “seroprevalence,” and “China” were used to search.

The search terms “cattle” (in Chinese) and “bluetongue” (in Chinese) were used for advanced search in the Chinese databases. All the Chinese databases used fuzzy search and synonym expansion. All the retrieved citations were imported into Endnote X9 (version 9.3.1).

Eligible studies were selected in accordance with the following criteria: (1) the objects of the research must be cattle; (2) the study aim must be to investigate the seroprevalence of bluetongue infection in cattle; (3) data must include information on the number of examined cattle and the number of bluetongue-positive cattle; (4) the study location must be in China; (5) the study design must be a cross-sectional study; and (6) the study must be published in Chinese or English. Studies that did not meet all the abovementioned criteria were excluded. Duplicate studies and review studies (not research papers) were also excluded.

### Data Extraction and Quality Assessment

Four trained reviewers performed the data extraction separately. Any differences in the process were determined by another reviewer (the author of this article). The following information was extracted from all the collected studies: first author, publication year, sampling year, geographical region of the study, cattle variety, total number of examined cattle, the number of bluetongue*-*positive cattle, diagnostic tests, and farming mode. To reduce the heterogeneity caused by the different detection methods, we only extracted the results of the serological detection methods [agar gel immunodiffusion (AGID) and enzyme-linked immunosorbent assay (ELISA)] in the study and always used ELISA as the primary detection method. When a study used both ELISA and AGID (or other methods), we extracted only the rate obtained by the ELISA method for subsequent data analysis. The database was established by Microsoft Excel (version 16.32).

We assessed the quality of the publications selected based on these criteria using a method derived from the Grading of Recommendations Assessment, Development, and Evaluation method (24–27). Studies were awarded one point each if they clearly introduced the testing methods, sampling method and time described in detail, whether or not sampling was random, and whether there were four or more potential risk factors. Studies with four or five points were deemed as high quality, those with two or three points were considered to be of moderate quality, and studies scoring zero or one point were marked as low quality.

We conducted this meta-analysis of proportions in R v3.5.2 (“R Core Team, R: A language and environment for statistical computing,” R Core Team 2018), where the “meta” package was used to estimate the models (28). When the seroprevalence is small or large, the variance becomes very small. As a result, this type of research tends to have more weightage in the meta-analysis. Therefore, we used transformation methods to avoid skewed distributions due to too large (close to 1) or too small (close to 0) seroprevalence in the included studies (29). Before the meta-analysis was performed, the proportions were converted using logarithmic conversion (PNL), logit transformation (PLOGIT), arcsine transformation (PAS), double-arcsine transformation (PFT), and no transformation (PRAW) (30). Then, a normal distribution test was carried out on the observed and transformation proportions. Judgment criteria were based on previous reports (29, 30). For reporting, the transformed summary proportion and its confidence interval were converted back to proportions for ease of interpretation (28). The following codes were used for this portion of the analysis:

**Table:** 

logarithmic conversion (PNL)	rate<-transform [m1, log=log(event/*n*)]; shapiro.test(rate$log)
logit transformation (PLOGIT)	rate<-transform{m1, logit=log[(event/*n*)/(1 – event/*n*)]}; shapiro.test(rate$logit)
arcsine transformation (PAS)	rate<-transform{m1, arcsin.size=asin[sqrt(event/ (*n* + 1))]}; shapiro.test(rate$arcsin)
double-arcsine transformation (PFT)	rate<-transform{m1,darcsin = 0.5*[asin(sqrt(event /(*n* + 1))) + asin((sqrt(event+1)/(*n* + 1)))]}; shapiro.test(rate$darcsin)
no transformation (PRAW)	rate<-transform[m1, r=event/*n*]; shapiro.test(rate$r)

High heterogeneity can be expected in the meta-analysis of seroprevalence. Therefore, we use the random-effect model in advance to perform overall data integration and subgroup analysis. We assessed the heterogeneity between studies using the *I*^2^ and Cochrane *Q* statistics (expressed in χ^2^ and *P*-values, respectively). *I*^2^ < 50% indicates low heterogeneity; *I*^2^ > 50% indicates high heterogeneity, which describes the percentage of differences between studies due to heterogeneity.

The visualized statistical results of the meta-analysis were represented by forest plots. The funnel plot and Egger's test were used to detect publication bias, and sensitivity analysis was used to verify the stability of results. Subgroup analysis and single-factor regression analysis were used to analyze heterogeneity. The following codes were used for this portion of the analysis:

**Table:** 

Forest plots	forest [meta1, xlim=c(−0.2, 0.8)]
Funnel chart	funnel (meta1)
Egger's test	metabias (meta1, method=“linreg”)
The sensitivity analysis	metainf (meta1, pooled = “random”) forest (metainf (meta1, pooled = “random”), xlim=c(0, 0.2))
Subgroup analysis	meta1<-metaprop(event, *n*, study, data=rate, sm=“PFT,” incr=0.5, allincr=TRUE, addincr=FALSE, title=“,” byvar= subgroup title, print.byvar=TRUE)
Meta-regression analysis	metareg (meta1, ~covariate title)

In the funnel diagram, the symmetry of the figure is judged subjectively. If it is symmetrical, there may be no publication bias or heterogeneity. If it is asymmetric, there may be publication bias or heterogeneity. According to the *P-*value, Egger's test was used to assess the publication bias of studies. The bias was considered to be non-existent when *P* ≥ 0.05. There may be publication bias if *P* < 0.05. In the sensitivity analysis, one study was deleted at a time and other studies were analyzed to estimate whether a single study would have a significant impact on the results.

Simultaneously, we conducted subgroup analysis stratified by the following potential risk factors: the investigated factors included the region (Northern China vs. other regions), sampling year (2012 or later vs. 2000 or before and 2001–2011), detection method [competitive (C)-ELISA vs. AGID], cattle variety (yak vs. buffalo, dairy cow, and yellow cattle), farming mode (comparison of intensive farming with free range), and study quality (high vs. middle and low). In the meta-analysis of seroprevalence, the detection method is usually the source of heterogeneity. Here, we used the detection method as a covariate and performed multivariate meta-regression analysis with other risk factors to explain the heterogeneity caused by the detection method.

We were not able to conduct subgroup analysis with the presence of mixed breeding with sheep or other ruminants as a covariate, as almost none of the studies we included mentioned this. We used zoogeographical division to conduct subgroup analysis and regression analysis on the included areas and conducted joint analysis with the distribution of the *Culicoides* species mentioned (31) to further enrich the potential risk factors affecting the seroprevalence of bluetongue disease in cattle in China.

## Results

**Figure 1:**
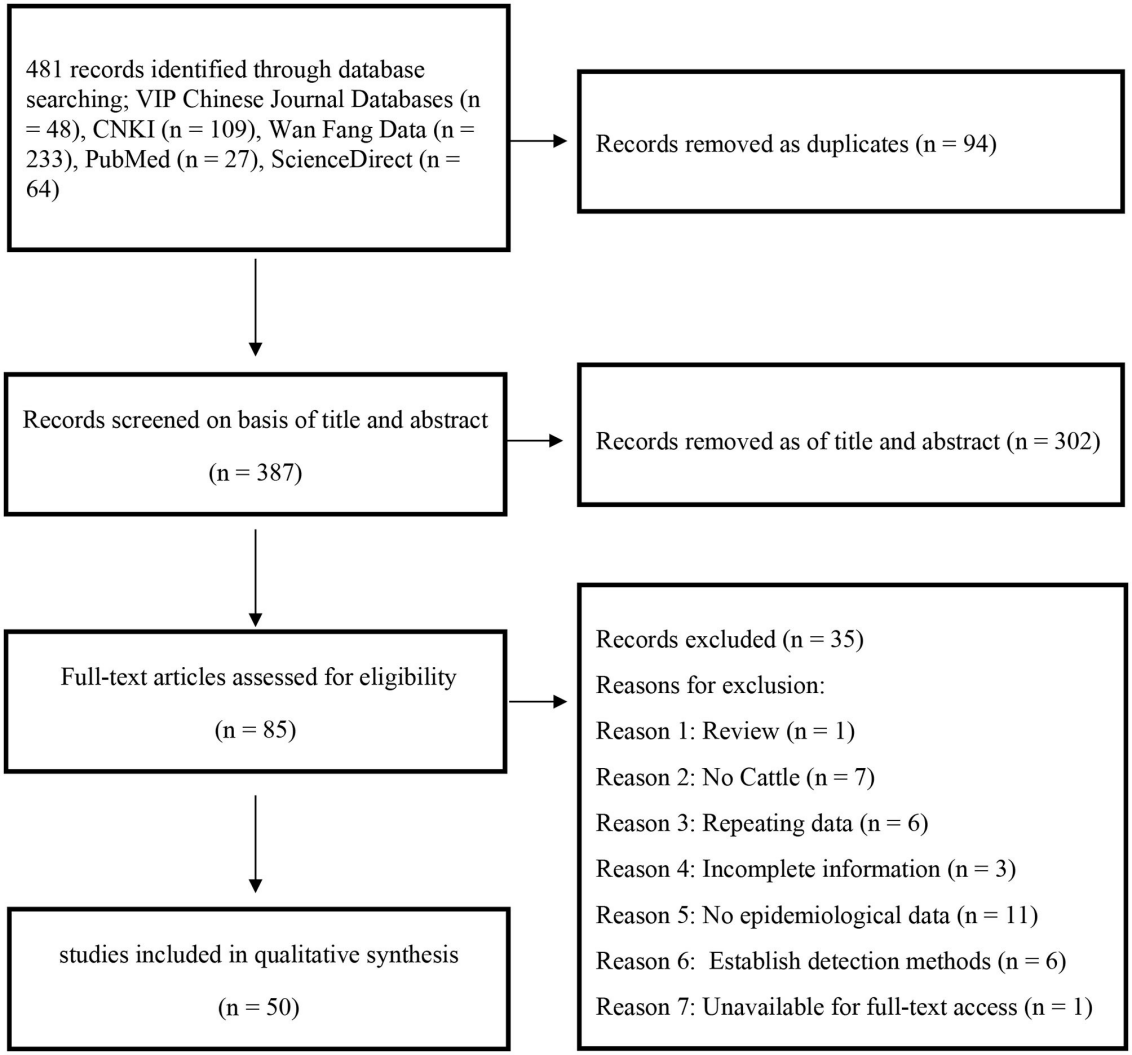
PRISMA chart of the study selection process showing inclusion and exclusion of studies.

In this study, 481 records were identified after searching five databases, and 85 papers were selected after the initial screening and removal of duplicates. An additional 35 articles were excluded for the following reasons: 1 was a review, 7 did not include cattle, 6 used repetitive data, 3 papers had incomplete information, 11 articles lack epidemiological data that could be extracted, 6 papers used established detection methods, and 1 article was unavailable for full-text access. Finally, 50 publications were used for the meta-analysis (Figure 1).

The *P* and *I*^2^ statistics demonstrated that a random-effects model should be used in this study (χ^2^ = 12,688.50, *I*^2^ = 99.6%, *P* = 0.00). A total of 87,472 cattle across seven regions and 23 provinces of China were investigated, and the results revealed that the pooled seroprevalence of bluetongue was 12.2% (5,332/87,472; 95% CI: 8.4–16.6) (Supplementary Figure 1, **Table 2**). According to our quality criteria, 5 papers were considered to be of high quality (four or five points), 34 were of moderate quality (two or three points), and the remaining 11 papers were deemed to be of low quality (zero to one point).

Four positive conversions were performed on the data (Table 1). The results showed that the conversion results of PAS and PFT may be closer to the normal distribution (30). According to Barendregt et al. (29), PFT can stabilize the variance more effectively. Finally, we chose the combination result of PFT conversion for meta-analysis (32).

**Table 1:** Normal distribution test for the normal rate and the different conversion of the normal rate.

	***W***	***P***
PRAW	0.796	7.106e−07
PLN	NaN	NA
PLOGIT	NaN	NA
PAS	0.921	0.003
PFT	0.918	0.002

*PRAW: original rate, PLN: logarithmic conversion, PLOGIT: logit transformation, PAS: arcsine transformation, PFT: double-arcsine transformation, NaN: meaningless number, NA: missing data*.

According to the funnel chart, we judged the existence of publication bias or heterogeneity in the selected study (Supplementary Figure 2). The results of Egger's test showed the following values: *t* = 4.582, *P* = 3.294e−05, indicating that there might be publication bias in the included studies (Supplementary Figure 3, Supplementary Table 1). We also used funnel plots for all the subgroups to assess publication bias (Supplementary Figures 4–9). The results showed that 35 studies did not clearly introduce whether to use random sampling, except for the 15 studies that explicitly mentioned random sampling. Therefore, our research may have sampling bias. The results of the sensitivity analysis indicated that the pooled seroprevalence was not significantly affected by any single study after omitting one study at a time; therefore, we believed that the results of our meta-analysis were reliable (Supplementary Figure 10).

**Figure 2:**
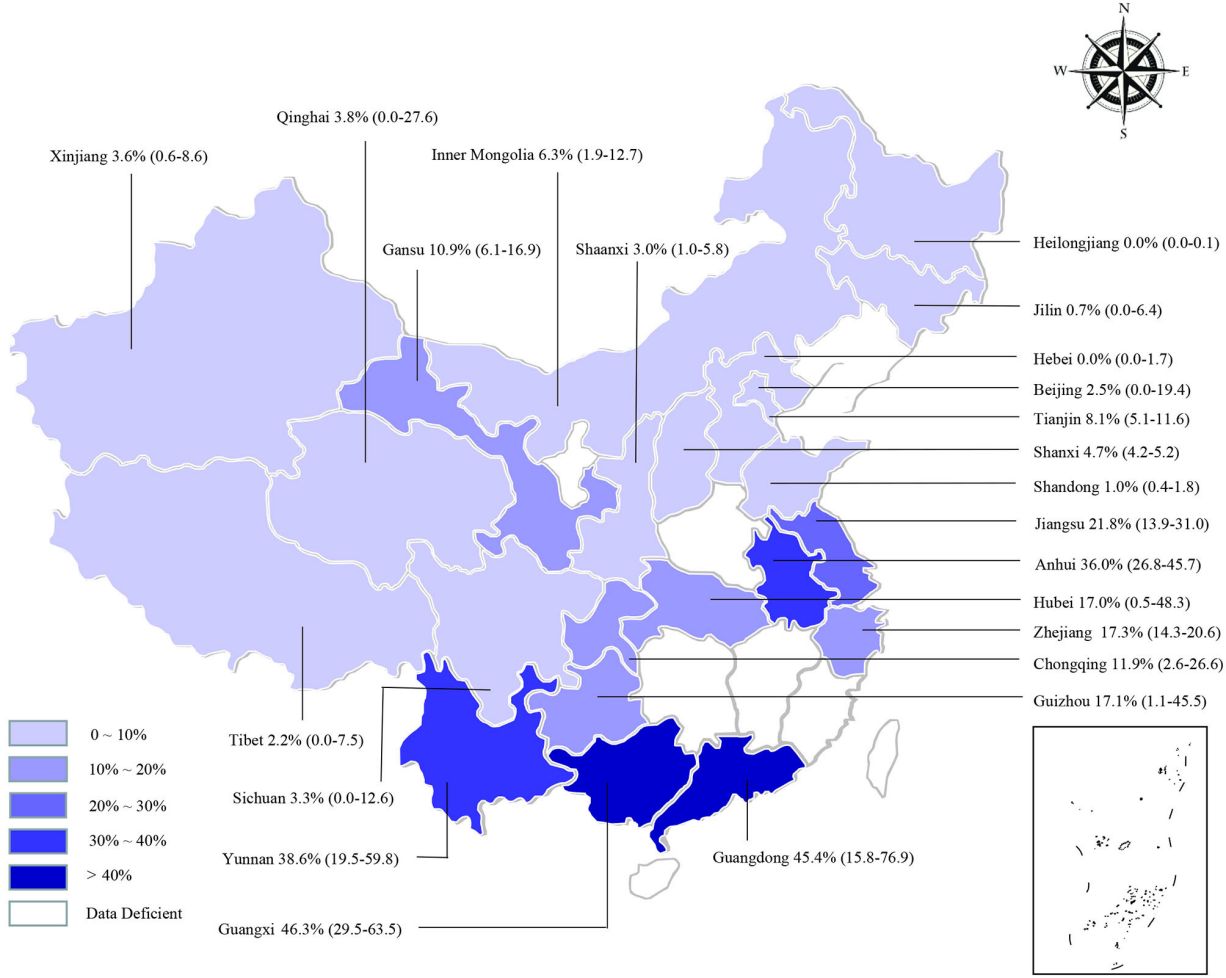
Bluetongue virus infection in cattle in China. Different saturations in the HSB slider represent different infection rates.

We estimated the potential risk factors including geographical distribution, sampling year, detection methods, species, farming mode, and study quality (**Table 3**). There was significantly high heterogeneity in all the subgroups, and all estimates of the pooled seroprevalence for each subgroup were calculated using the random-effects model. In terms of geographical region, the highest seroprevalence of bluetongue was 45.9% (95% CI: 30.9–61.4; 1,289/3,178) in Southern China and the lowest seroprevalence was 0.1% (95% CI: 0.0–0.9; 4/3,467) in Northeastern China, and the difference was statistically significant (*P* < 0.05). We obtained preliminary statistics on the seroprevalence of bluetongue in different provinces. The results showed that the highest positive rate of bluetongue was 46.3% (95% CI: 29.5–63.5) in Guangxi Province, followed by Guangdong at 45.4% (95% CI: 15.8–76.9). The seroprevalence of bluetongue in Hebei (0.0%; 95% CI: 0.0–1.7), Heilongjiang (0.0%; 95% CI: 0.0–0.1), and Jilin (0.7%; 95% CI: 0.0–6.4) was lower than that in other provinces (Figure 2, Table 2).

**Table 2:** Included studies of bluetongue virus in cattle.

	**Province**	**Detection method**	**Positive samples/total samples**	**Study quality**
Zhang and Zheng (33)	Hubei	AGID	61/979	Middle
Wang et al. (34)	Hubei	C-ELISA	64/200	Middle
Mao et al. (35)	Jiangsu	C-ELISA	65/369	Middle
Zhu et al. (36)	Jiangsu	AGID	97/368	Middle
Jiao (37)	Anhui	AGID	36/100	Middle
Lin (38)	Shandong	C-ELISA	7/737	Low
Wang and Zhao (39)	Zhejiang	AGID	97/560	Low
**Northeastern China**				
Zhong et al. (40)	Heilongjiang	C-ELISA	0/1,594	High
Meng et al. (41)	Jilin	AGID	0/1,738	Low
Wang et al. (34)	Jilin	C-ELISA	4/135	Middle
**Northern China**				
Chen et al. (42)	Shanxi	C-ELISA	31/592	Middle
Lv (43)	Inner Mongolia	C-ELISA	104/796	Middle
Zhang (44)	Inner Mongolia	C-ELISA	0/110	Middle
Liang and Zhang (45)	Shanxi	AGID	281/6,059	Low
Li and Li (46)	Beijing	C-ELISA	61/652	Low
Li and Li (46)	Tianjin	C-ELISA	22/273	Low
Zhu and Li (47)	Beijing	AGID	0/3,248	Middle
Han (48)	Inner Mongolia	C-ELISA	60/472	Middle
Wang et al. (34)	Inner Mongolia	C-ELISA	5/100	Middle
Wang et al. (34)	Hebei	C-ELISA	0/100	Middle
Lin (49)	Xinjiang	AGID	415/45,018	Low
Zhang et al. (50)	Shaanxi	AGID	9/548	Middle
Liang and Lin (51)	Shaanxi	AGID	296/6,639	Middle
He (52)	Qinghai	AGID	0/204	Middle
Shi et al. (53)	Xinjiang	C-ELISA	0/250	Middle
Ma et al. (54)	Gansu	C-ELISA	211/1,584	High
Wang (55)	Xinjiang	C-ELISA and AGID	39/1,251	Middle
Nu et al. (56)	Xinjiang	C-ELISA	4/96	Middle
Bai et al. (57)	Gansu	AGID	8/108	Middle
Bai et al. (57)	Qinghai	AGID	9/65	Middle
Bai et al. (57)	Shaanxi	AGID	2/51	Middle
Zhao and Tao (58)	Xinjiang	C-ELISA	0/180	Middle
Wang et al. (34)	Xinjiang	C-ELISA	62/176	Middle
**Southern China**				
Lv et al. (59)	Guangdong	C-ELISA	362/520	High
Lin et al. (60)	Guangxi	AGID	216/496	High
Lin et al. (61)	Guangxi	AGID	184/417	Middle
Li et al. (62)	Guangxi	AGID	92/387	Middle
Deng and Peng (63)	Guangdong	AGID	250/1,042	Middle
Huang (64)	Guangdong	AGID	71/164	Middle
Wang et al. (34)	Guangxi	C-ELISA	114/152	Middle
**Southwestern China**				
Zhu (65)	Yunnan	AGID	148/400	Middle
Zhang and An (66)	Sichuan	C-ELISA	0/590	Middle
Yuan et al. (67)	Chongqing	C-ELISA	10/160	Middle
Luo et al. (68)	Guizhou	AGID	10/447	Low
Li et al. (69)	Yunnan	C-ELISA	113/220	Middle
He et al. (70)	Guizhou	C-ELISA	190/503	Middle
Kong et al. (71)	Yunnan	C-ELISA	28/39	High
Wei et al. (72)	Yunnan	AGID	232/478	Middle
Luo et al. (73)	Guizhou	AGID	3/90	Low
Yun et al. (74)	Yunnan	C-ELISA	420/540	Middle
Cao (75)	Yunnan	C-ELISA	89/627	Low
Han et al. (76)	Tibet	C-ELISA	49/674	Middle
Qu and Gao (77)	Tibet	C-ELISA	0/739	Middle
Suo et al. (78)	Tibet	AGID	4/514	Middle
Cao et al. (79)	Yunnan	AGID	55/417	Low
Xiao et al. (80)	Sichuan	C-ELISA	28/221	Low
Xiao et al. (80)	Yunnan	C-ELISA	11/76	Low
Li et al. (81)	Sichuan	C-ELISA	15/511	Middle
Li et al. (81)	Tibet	C-ELISA	11/225	Middle
Duan et al. (82)	Yunnan	C-ELISA	20/507	Middle
Wang et al. (34)	Chongqing	C-ELISA	45/240	Middle
Wang et al. (34)	Guizhou	C-ELISA	46/110	Middle
Wang et al. (34)	Yunnan	C-ELISA	466/614	Middle

*C-ELISA, competitive enzyme-linked immunosorbent assay; AGID, agar gel immunodiffusion*.

Furthermore, the pooled seroprevalence of bluetongue was 16.6% (95% CI: 9.6–25.0; 1,922/22,348) in cattle collected before 2000, 22.5% (95% CI: 1.2–58.9; 400/1,117) in cattle collected during 2001–2011, and 9.9% (95% CI: 3.3–19.4; 1,976/12,564) in cattle collected in 2012 or later. The estimated pooled seroprevalence of bluetongue was significantly higher (*P* < 0.05) among free-range cattle at 22.5% (95% CI: 7.7–42.3; 210/1,002) than among intensive farming cattle at 1.8% (95% CI: 0.0–6.7; 136/7,497). The pooled seroprevalence of bluetongue in different species showed significant variation (*P* < 0.05), with the highest seroprevalence of 39.8% (95% CI: 18.7–63.0; 716/1,675) in buffalo and the lowest seroprevalence of 4.3% (95% CI: 1.2–9.0; 289/3,822) in yak. The seroprevalence of bluetongue investigated by AGID was 12.2% (95% CI: 7.6–17.8; 2,764/71,300), and in the C-ELISA test, it was 11.6% (95% CI: 5.8–19.1; 2,568/16,172), and no significant difference was found (*P* = 0.717) (Table 3). In addition, joint analysis of the detection method and other subgroups showed that the detection method had the greatest impact on the farming mode subgroup [*R*^2^ = 31.47%; residual variation due to homogeneity (*I*^2^-res) = 99.04%] and had the least impact on the research quality (*R*^2^ = 0%; *I*^2^-res = 99.51%) (Table 3). In the zoogeographical division subgroup, the Southern China district had the highest disease incidence (47.5%, 42.1–52.9%), which corresponded with the most abundant species of *Culicoides* (*n* = 129).

**Table 3:** Pooled prevalence of bluetongue virus in cattle.

		**No. of studies**	**No. of tested**	**No. of positive**	**% (95% CI)**	**Heterogeneity**			**Univariate meta-regression**			
						**χ*2***	***P*-value**	***I2* (%)**	***P*-value**	**Coefficient (95% CI)**	***R*^**2**^ (%)**	***I*^**2**^-res (%)**
Region*									0.023	0.202 (0.028 to 0.377)	25.56	99.16
	Eastern China	5	2,134	302	17.1% (5.4–33.4)	295.85	<0.010	98.6				
	Southern China	7	3,178	1,289	45.9% (30.9–61.4)	446.66	<0.010	98.7				
	Northwestern China	11	56,170	1,055	4.3% (2.0–7.4)	999.46	<0.010	98.8				
	Central China	2	1,179	125	17.0% (0.6–48.3)	81.09	<0.010	98.8				
	Southwestern China	19	8,942	1,993	18.8% (8.8–31.5)	4,150.22	<0.010	99.5				
	Northern China	8	12,402	564	4.0% (1.1–8.6)	675.23	<0.010	98.8				
	Northeastern China	3	3,467	4	0.1% (0.0–0.9)	15.12	<0.010	86.8				
Sampling year									0.184	0.112 (−0.053 to 0.276)	1.38	99.53
	2000 or before	18	22,348	1,922	16.6% (9.6–25.0)	3,552.52	0.000	99.5				
	2001 to 2011	3	1,117	400	22.5% (1.2–58.9)	316.93	<0.010	99.4				
	2012 or later	18	12,564	1,976	9.9% (3.3–19.4)	3,987.60	0.000	99.6				
Detection method									0.869	0.010 (−0.111 to 0.131)	5.92	99.55
	AGID	24	71,300	2,764	12.2% (7.6–17.8)	6,274.78	0.000	99.6				
	C-ELISA	27	16,172	2,568	11.6% (5.8–19.1)	4,580.41	0.000	99.4				
Variety									0.016	0.313 (0.058 to 0.568)	24.50	98.65
	Buffalo	7	1,675	716	39.8% (18.7–63.0)	476.83	<0.010	98.7				
	Dairy cow	9	5,318	399	15.3% (3.4–33.2)	1,264.57	<0.010	99.4				
	Yak	7	3,822	289	4.3% (1.2–9.0)	181.51	<0.010	96.7				
	Yellow cattle	11	1,559	303	24.4% (13.4–37.3)	273.81	<0.010	96.3				
Farming mode									0.005	0.356 (0.108 to 0.605)	31.47	99.04
	Free range	2	1,002	210	22.5% (7.7–42.3)	55.77	<0.010	96.4				
	Intensive farming	6	7,497	136	1.8% (0.0–6.7)	693.36	<0.010	99.1				
Study quality									0.009	−0.279 (−0.489 to −0.070)	0.00	99.51
	Low	11	56,915	1,079	5.5% (2.7–9.2)	1,121.36	<0.010	99.1				
	Middle	34	26,324	3,436	12.3% (7.0–18.9)	6,843.24	0.000	99.5				
	High	5	4,233	817	33.4% (6.4–68.4)	1,912.97	0.000	99.8				
Total		50	87,472	5,332	12.2% (8.4–16.6)	12,688.50	0.000	99.6				

Region*: Northeastern China: Heilongjiang and Jilin; Northern China: Shanxi, Inner Mongolia, Hebei, Tianjin, and Beijing; Northwestern China: Xinjiang, Qinghai, Gansu, and Shaanxi; Eastern China: Shandong, Jiangsu, Zhejiang, and Anhui; Central China: Hubei; Southern China: Guangdong and Guangxi; Southwestern China: Guizhou, Tibet, Yunnan, Sichuan, and Chongqing.

*R^2^: Proportion of between-study variance explained, I^2^-res: residual variation due to heterogeneity*.

Regression analysis showed that the region, cattle variety, farming mode, and quality score subgroup might be the main sources of heterogeneity (*P* < 0.05).

## Discussion

BTV infection should not be ignored because it causes significant economic losses to global livestock breeding. To our knowledge, this is the first meta-analysis of bluetongue seroprevalence in cattle in China. The findings of this study may provide control measures that can be implemented in the development of animal husbandry. The seroprevalence of bluetongue exhibited variations depending on differences in the region of investigated cattle, cattle variety, sampling year, and farming mode in the present analysis. The pooled seroprevalence of bluetongue in cattle was 12.2% (95% CI: 8.4–16.6), with statistical significance among different regions (Table 3). We found that the seroprevalence was higher in Guangdong, Guangxi, Yunnan, and Anhui and lower in Heilongjiang, Jilin, Xinjiang, and Tibet (Figure 2). The BTV transmission dynamics strongly depends on the local context such as the species present, their density and distribution, and climatic conditions (83). Guangdong, Guangxi, and Yunnan are neighboring provinces. It is worth noting that the geographical distribution was uneven, and the number of studies in some regions was very small in the researches included (Central China, *n* = 2; Northeastern China, *n* = 3), which may lead to unstable results. In addition to climatic factors, further cross-province circulation of animals and animal products should be verified. The present study confirms the widespread seroprevalence of bluetongue in cattle in China (Figure 2).

In the detection method subgroups, two detection methods were included (AGID and C-ELISA). Diagnostic technology for bluetongue disease of China (GB/T 18636-2017) stipulates that AGID and C-ELISA are suitable for detecting BTV antibody in serum samples. Since the establishment of the 2002 standard, reverse transcription–polymerase chain reaction (RT-PCR) and fluorescent RT-PCR molecular biological detection methods have been added. Molecular biology is currently the best detection method, with high sensitivity and strong specificity. RT-PCR methods were able to detect representative strains of all 24 BTV serotypes and different BTV serotypes in the Mediterranean region, with a detection limit of < 0.01 ECE_50_ (median effective dose) (84, 85). However, false-positive results are easy to occur because of contamination by nucleic acids using molecular biological methods with high sensitivity. Moreover, the high price of the instruments and the operation steps of replication limit the technology popularization; serological detection still dominates large-scale clinical testing (86). AGID is relatively simple and economical, but the antibodies in this method exhibit cross-reactivity with epizootic hemorrhagic disease virus, causing false-positive results (87). C-ELISA is a rapid method detecting antibodies in serum samples as early as day 6 post-infection (88) and has been widely commercialized. Based on the analysis of the sampling years, it showed that the seroprevalence of bluetongue in cattle detected pre-2000 was lower than that detected in 2001–2011. However, only three articles reported the detection methods among the studies from 2001 to 2011, and insufficient grouping data may lead to deviation from the actual situation. In addition, different detection methods may cause differences in the seroprevalence of bluetongue disease. AGID was the main detection method before 2000, and C-ELISA was the main detection method in 2001–2011. After China joined in the World Trade Organization (WTO) (89), its internationalization may have led to significant changes in China's animal husbandry. Although China joining the WTO was conducive to the large-scale industrialization of animal husbandry in China, the prevention and control of diseases by breeders was not consistent with the rapid expansion of production scale and the growth of demand (90), causing a rapid increase in the bluetongue infection rate among cattle from 2001 to 2011. We suggested that a comprehensive surveillance system should be established to improve the technical level of breeding management, so that the level of disease prevention and control can be developed together with the scale of breeding to avoid the wide seroprevalence of bluetongue. In the 2012 or later group, the detection method was mainly based on C-ELISA. Cattle collected from after 2012 had a lower seroprevalence of bluetongue than those collected from 2001 to 2011, indicating that the seroprevalence of bluetongue disease declined in recent years. This result may be related to the medium- and long-term animal disease prevention plan (2012–2020) issued by China in 2012, which strengthened animal disease prevention and control measures (91). It is necessary to continue implementing effective control regulations to reduce bovine bluetongue infection.

Our findings suggested that cattle variety (*P* = 0.015) may be the source of heterogeneity (Table 3). The seroprevalence of bluetongue was significantly higher in buffalo than in yellow cattle, yak, and cow, which may be due to potential vector preference for a host species (92). Moreover, it should be noted that both host susceptibility and climate variations between regions have a direct impact on vector distribution. As previously demonstrated, climate variables play a substantial role in promoting or hampering the development of bluetongue (93–96). The buffalo breeding area has a humid subtropical and temperate climate with an average temperature of 12–29°C and a rainfall of 800–1,500 mm. Therefore, the climatic and geographical conditions of the breeding area are conducive to the survival, propagation, and transmission of the *Culicoides* midges, which are the vectors that transmit BTV. A higher intensity of vector is directly linked with the increased seroprevalence of BTV (97). The zoogeographical division subgroup showed that the seroprevalence of bluetongue disease in cattle from China was correlated positively with the distribution of *Culicoides* species. Farms should pay attention to disseminating media such as *Culicoides* to reduce the spread of bluetongue disease in cattle. In addition, the seroprevalence of BTV among yak was the lowest, which may be related to the yak's habitat. It is mainly distributed in intermountain basins, alpine grasslands, and alpine desert grasslands of Xinjiang, Tibet, and Sichuan. This result is consistent with the trend of seroprevalence.

In different regions, with global warming, there is an indication that the prevention and control of bluetongue in cattle and other animals should be paid more attention. BTV infection may exhibit annual variation due to climatic modulation (98). Moreover, our findings suggested that the seroprevalence of bluetongue was lower in dairy cows than in yellow cattle. We conducted multivariate meta-regression analysis on varieties using regions as the covariates, and the results showed that regions were not a source of heterogeneity in the subgroup (*P* > 0.05, data not shown). Then, we carried out multivariate meta-regression analysis on varieties with years as covariates, and the results revealed that years were not the source of heterogeneity in the subgroup (*P* > 0.05, data not shown). We speculated that the difference in the seroprevalence between cows and yellow cattle may be caused by different breeding modes. However, data regarding the farming modes was insufficient for multivariate meta-regression analysis on varieties and farming modes. In general, dairy cows are mostly under the intensive farming system, while yellow cattle are mostly in the free-range system as draft cattle or beef cattle. This result is consistent with the farming mode subgroup.

The results revealed a significant difference in the seroprevalence of bluetongue between cattle in the free-range and intensive farming systems in the farming mode subgroup, indicating that risk behaviors do differ between farming mode. Intensive farming greatly reduced the infection rate of bluetongue disease, indicating that intensive farming had more advanced technologies (different feeding and management schemes are formulated for different animals, seasons, and ages; and strict disease prevention and control measures are taken) and better management in this regard. Moreover, cattle from the free-range farming system had a larger range of activities and more likely to be bitten by mosquitoes transmitting the BTV (83). Therefore, it is necessary to further strengthen intensive farming practices to control the domestic epidemic of bluetongue.

We tried to determine season as a potential risk factor, but results could not be obtained due to lack of adequate data. However, we speculated that season may be one of the potential risk factors affecting the change in seroprevalence, because warm temperature and human environment are positively associated with animal habitats. In fact, a 1°C increase in temperature has been reported to increase the risk of bluetongue by 27% (99). Optimal temperature is favorable for many processes of BTV transmission, such as incubation period, carrier capacity (100), and survival rate (101). Further studies are needed to confirm that there are no other influential factors, such as specific sampling time or season and sampling limitations.

In addition, we found that the study quality was the source of heterogeneity in the present study (*P* = 0.009), suggesting that more risk factors should be collected as quality evaluation.

There are some limitations in our meta-analysis that may affect the results. First, we found a number of potentially relevant studies through our systematic review, but not all the underlying studies were suitable for use. Therefore, some of these studies might not have been included in this meta-analysis, and some potential risk factors may be missed. Second, numerous qualified studies were acquired in our systematic review, but not all data were available. There is not enough data for the subgroup analysis on the seroprevalence of bluetongue, such as the breeding model and variety. Third, analyzable data were limited, involving age, sex, living environment, the presence or absence of sheep or other ruminants, the presence or absence of *Culicoides* or other vectors, and season. We correlated the relationship between *Culicoides* species diversity and zoogeographical divisions in China; however, as far as the authors know, only a few types of Kumon can transmit BTV, so the results of this part should be treated with caution. Researchers should conduct an extensive survey of the distribution of BTV vectors to further study the relationship between BVT infection and transmission vectors. All these variates were not analyzed in our study. The breeding conditions might lead to differences, which is probably the result of differences in environmental conditions. Fourth, a limited number of qualifying researches in Central China (*n* = 2) and Northeastern China (*n* = 3) might not reflect the true seroprevalence in the investigated regions, and the quality level of the study was variable, suggesting that more surveillance of bluetongue infection should be continuously undertaken in cattle in these areas. Fifth, some of the included studies did not explicitly mention whether to use random sampling. Therefore, our research may have a sampling bias. Sixth, though the studies included in this review were eligible, most were of moderate or low quality. This may be due to the lack of the underlying factor of sampling randomness or sampling method. To prove the quality of research, more risk factors should be considered and analyzed in the future.

Based on a systematic meta-analysis, we assessed the seroprevalence of bluetongue in cattle from China. The results showed that bluetongue was epidemic in cattle in China. Region, variety, feeding methods, and other factors might affect the seroprevalence of bluetongue. We suggested that appropriate control schemes be formulated according to the differences in breeding patterns and geographical conditions in the various regions. It is also necessary to conduct epidemiological investigations on cattle in more regions to further explore the risk factors of bluetongue infection in cattle. Comprehensive surveillance programs should be adopted to prevent the spread of bluetongue in cattle.

## Data Availability Statement

The original contributions presented in the study are included in the article/Supplementary Materials, further inquiries can be directed to the corresponding author/s.

## Author Contributions

FL and RD are responsible for the idea and concept of the paper. D-LL, BZ, G-YG, and J-ML collected the data. QW, YZ, and X-YY analyzed the results. Q-LG wrote the manuscript. XL and KS revised the manuscript. All the authors contributed to the manuscript editing and approved the final manuscript.

## Conflict of Interest

The authors declare that the research was conducted in the absence of any commercial or financial relationships that could be construed as a potential conflict of interest.

## Abbreviations

AGID: agar gel immunodiffusion
BTV: bluetongue virus
CNKI: Chinese Web of Knowledge
OIE: World Organization for Animal Health
WTO: World Trade Organization.
