# Supplementary material for: Seroprevalence and Risk Factors of the Bluetongue Virus in Cattle in China From 1988 to 2019: A Comprehensive Literature Review and Meta-Analysis
Source: Front Vet Sci. 2021 Jan 28;7:550381. doi: 10.3389/fvets.2020.550381 (PMC7901971; doi:10.3389/fvets.2020.550381)
Supplement: Supplementary file 1 [file Data_Sheet_1.docx]

**Table S1.**Egger’s for Publication Bias

| slope | bias | se.bias | t | df | p-value |
| --- | --- | --- | --- | --- | --- |
| 0.084 | 12.287 | 2.686 | 4.582 | 48 | 3.294e-05 |

**TableS2** BTV infection in cattle and*Culicoides* spp.distributioninzoogeographical region in China

| Zoogeographical region | % (95% CI*) | *No. Culicoides*spp. |
| --- | --- | --- |
| Northeastern China district | 0.30% (0.6-6.6) | 66 |
| Northern China district | 18.8% (11.1-32.9) | 48 |
| Southern Chinadistrict | 47.5% (42.1-52.9) | 129 |
| CentralChinadistrict | 17.1% (8.6-27.6) | 71 |
| Inner Mongolia-Xinjiang district | 5.1% (1.5-10.6) | 55 |
| Qinghai-Tibet district | 3.5% (0.6-8.3) | 23 |
| Southwestdistrict | 8.9% (4.9-13.8) | 82 |


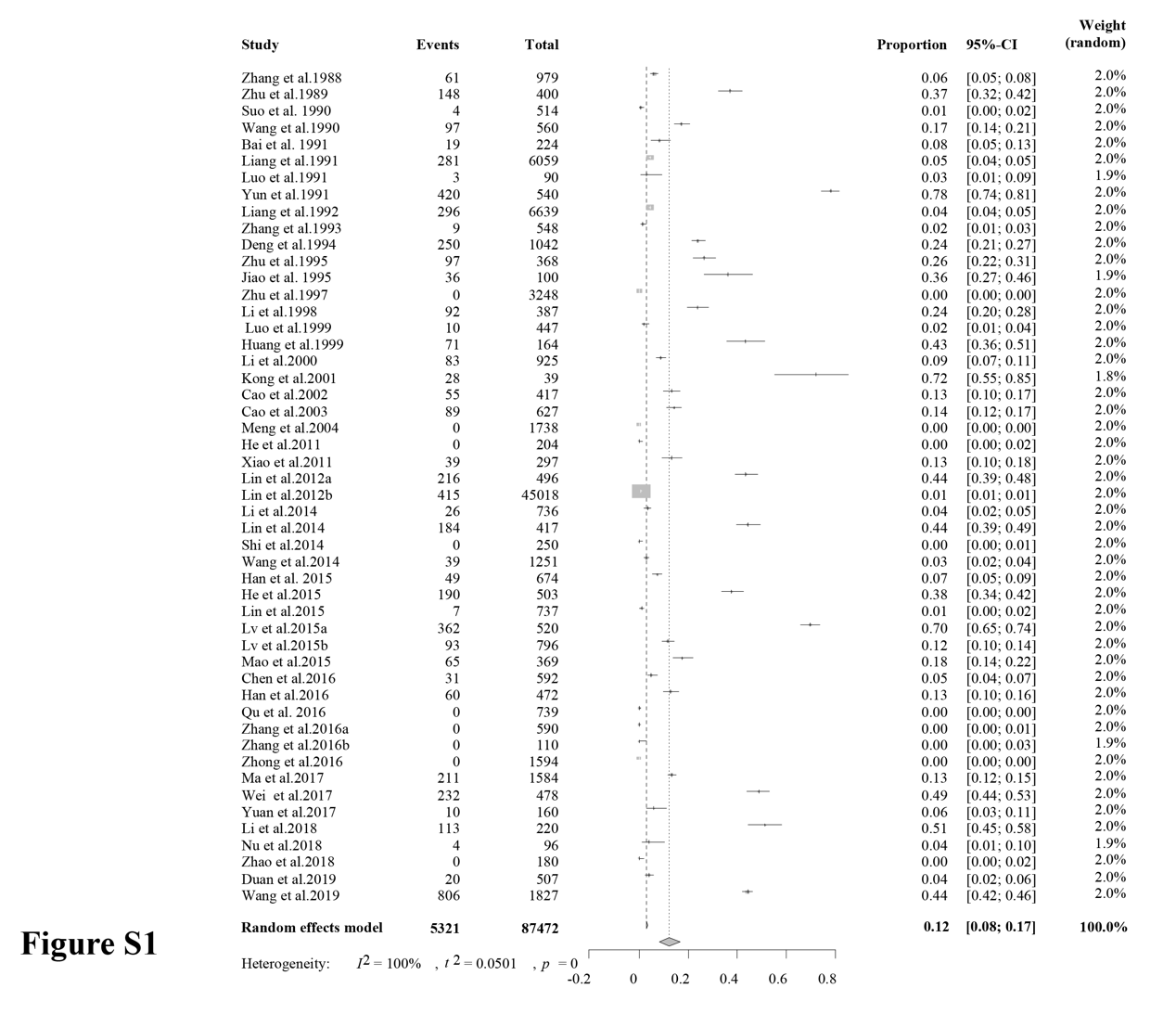


**FigureS1.**Forest plot of bluetongue prevalence among studies conducted in China. The length of the horizontal line represents the 95% CI; the diamond represents the summarized effect


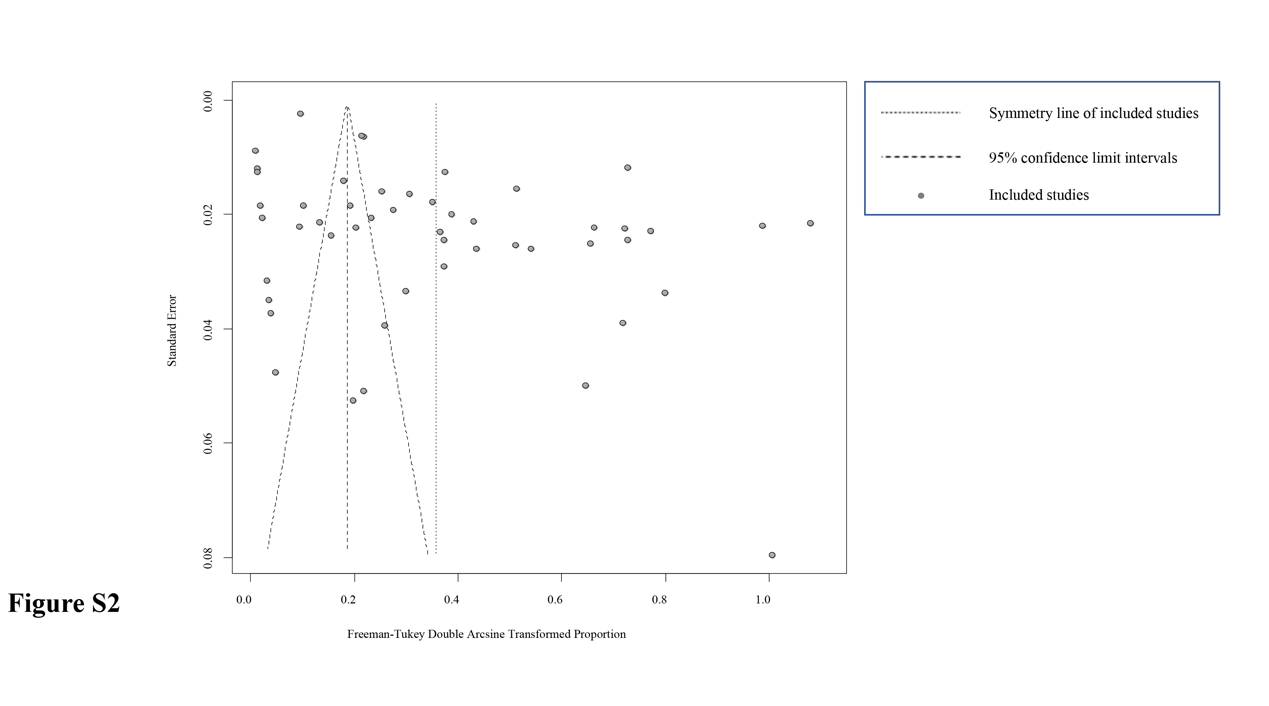


**FigureS2.** Funnel plot with pseudo 95% confidence limit intervals to examine publication bias

0

100

200

300

400

0

10

20

30

40

50

60

Inverse of standard error

Standardised treatment effect (z-score)

**Figure S3**The Egger’s publication bias plot

**Figure S4.** Funnel plot with pseudo 95% confidence limits intervals for the examination of publication bias of region subgroup

**Figure S5.** Funnel plot with pseudo 95% confidence limits intervals for the examination of publication bias of sampling year subgroup

**Figure S6.** Funnel plot with pseudo 95% confidence limits intervals for the examination of publication bias of detection method subgroup

**Figure S7.** Funnel plot with pseudo 95% confidence limits intervals for the examination of publication bias of variety subgroup

**Figure S8.** Funnel plot with pseudo 95% confidence limits intervals for the examination of publication bias of farming mode subgroup


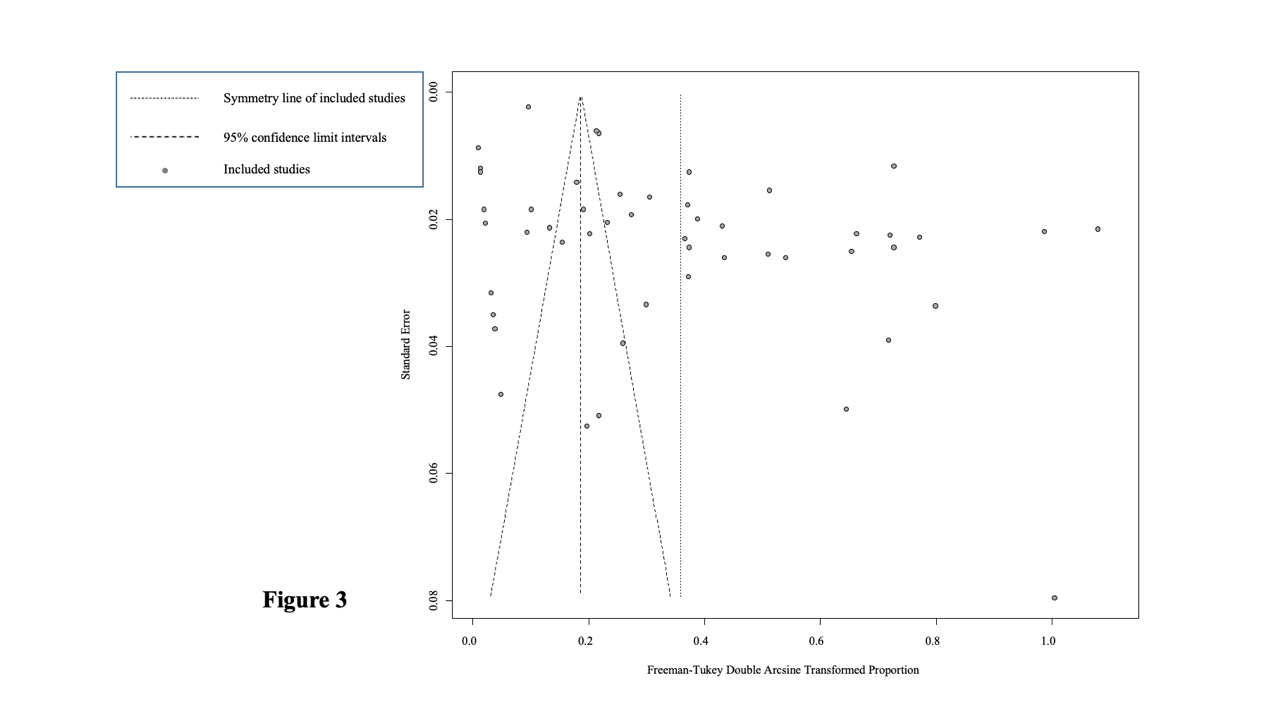
**Figure S9.** Funnel plot with pseudo 95% confidence limits intervals for the examination of publication bias of study quality subgroup


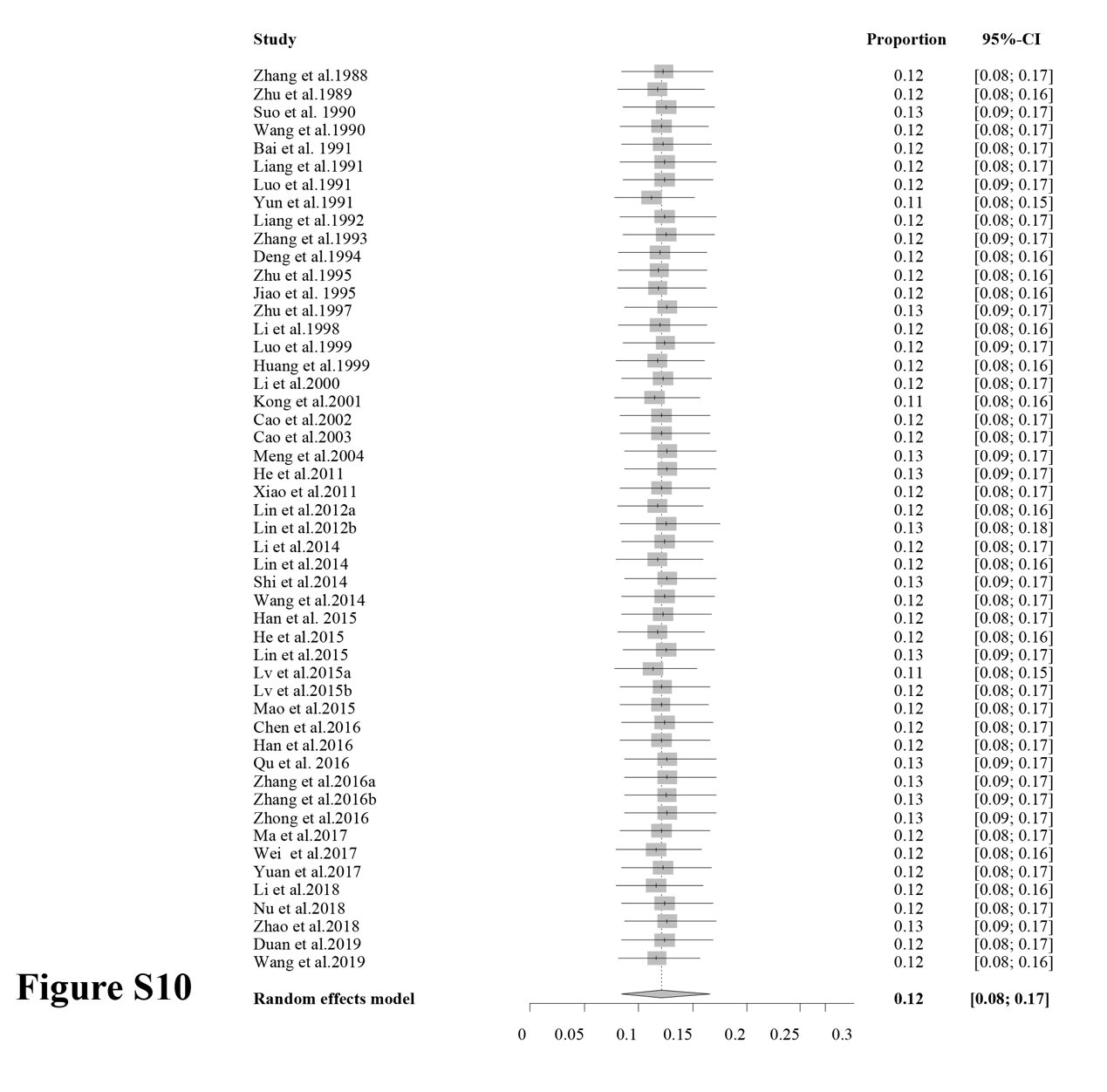


**Figure S10.** Sensitivity analysis. After removing one study at a time, the remaining studies were re-combined using a random-effects model to verify the impact of a single study on the overall results
